# A Circuit-Level Solution for Secure Temperature Sensor

**DOI:** 10.3390/s23125685

**Published:** 2023-06-18

**Authors:** Mashrafi Alam Kajol, Mohammad Mezanur Rahman Monjur, Qiaoyan Yu

**Affiliations:** Department of Electrical and Computer Engineering, University of New Hampshire, Durham, NH 03824, USA; mashrafialam.kajol@unh.edu (M.A.K.); mohammad.monjur@unh.edu (M.M.R.M.)

**Keywords:** hardware security, battery thermal management system, temperature sensor, under-powering attack, hardware Trojan, anomaly injection

## Abstract

Temperature sensors play an important role in modern monitoring and control applications. When more and more sensors are integrated into internet-connected systems, the integrity and security of sensors become a concern and cannot be ignored anymore. As sensors are typically low-end devices, there is no built-in defense mechanism in sensors. It is common that system-level defense provides protection against security threats on sensors. Unfortunately, high-level countermeasures do not differentiate the root of cause and treat all anomalies with system-level recovery processes, resulting in high-cost overhead on delay and power consumption. In this work, we propose a secure architecture for temperature sensors with a transducer and a signal conditioning unit. The proposed architecture estimates the sensor data with statistical analysis and generates a residual signal for anomaly detection at the signal conditioning unit. Moreover, complementary current–temperature characteristics are exploited to generate a constant current reference for attack detection at the transducer level. Anomaly detection at the signal conditioning unit and attack detection at the transducer unit make the temperature sensor attack resilient to intentional and unintentional attacks. Simulation results show that our sensor is capable of detecting an under-powering attack and analog Trojan from a significant signal vibration in the constant current reference. Furthermore, the anomaly detection unit detects anomalies at the signal conditioning level from the generated residual signal. The proposed detection system is resilient against any intentional and unintentional attacks, with a detection rate of 97.73%.

## 1. Introduction

Sensors have been widely applied to various applications, such as factory automation [1] and Internet-of-Things [2,3]. It is predicted that the global sensors market size will grow from USD 204.80 billion in 2022 to around USD 508.64 billion by 2032 [4]. Among different types of sensors, a temperature sensor detects and measures coldness and heat and then converts its measurement into an electrical signal, which can facilitate automatic process control [5], quality inspection [6], and hazard management [7].

**Figure 1:**
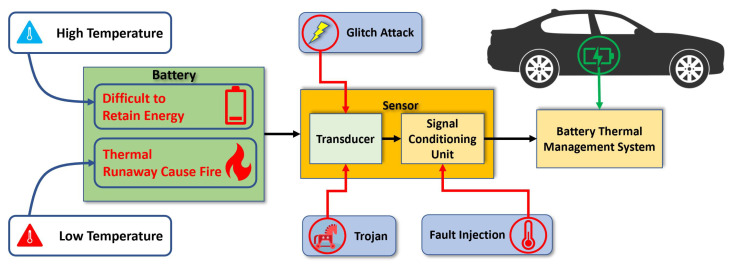
Threat analysis for temperature sensors deployed to an electric vehicle.

As sensors are typically low-end devices, the security issues on sensors are not considered as a high priority. Unfortunately, due to the important role of sensors in safety-critical applications, it is imperative to assure the integrity, reliability, and security of temperature sensors. For example, the safety and dependability of EVs may be jeopardized if temperature sensors are exposed to security risks. The threat analysis of the temperature sensor for electric vehicles (EVs) lithium-ion batteries is shown in Figure 1. To mitigate the problems related to temperature, EVs have one dedicated section called battery thermal management systems (BTMS) [8]. This system analyzes all thermal sensors’ data from the environment surrounding the battery and manages the thermal runaway. Temperature sensors serve as a bridge between physical quantities surrounding the battery and the BTMS. Identifying sensor malfunctions in the BTMS as quickly as possible is crucial since they might have serious negative impacts on the system.

Due to limited resources to authenticate the source of signals, sensors typically cannot tolerate intentional or unintentional interference (such as a fault attack) [9]. Security threats on sensors are typically managed at the system level. Sensor fusion [10] relies on system-level statistics to detect anomalies in the overall system. Fuzz testing and validated security patches capture the system anomalies, but they cannot identify a transduction attack that provides false sensing data [11]. A sensor network redundant system [12] is introduced to detect the inconsistency among multiple sensors. A structural analysis-based sensors fault detection method [13] generates sequential residuals to detect and isolate current, voltage, and temperature sensor faults. The residuals are evaluated by a statistical inference method for more accurate decisions. A study [14] proposed a sensor fault detection strategy based on a data-driven method and optimized by utilizing five different machine learning methods. Although these systems can accurately detect stealthy attacks with a short detection time, sensor-level detection is still needed to identify the attack location. System-level solutions do not differentiate the root causes and thus treat various attacks with the same recovery procedure, sometimes incurring unnecessary overhead. Attack mitigation from a high level will fail to meet the real-time requirement of practical applications. Moreover, some existing works designed temperature sensors only assuring reliability at the circuit level. A transistor-based current-mode thermal sensor [15] leverages subthreshold NMOS transistors to meet power requirements with a robust architecture. To mitigate the sensitivity to the subthreshold factor variability, a simple voltage-based single-point soft-trimming was implemented. A complimentary current-mode approach [16] using a single feedback loop is introduced to design a compact thermal sensor. A fully integrated temperature sensor [17] utilized the difference between a reference current source and a proportional to absolute temperature current source to generate a linear temperature-dependent frequency. The reliability of this design is ensured by using a process compensator switch to the architecture. A recent work [18] presented a bandgap reference voltage source for a sensor system-on-chip by combining the high stability of the traditional BJT bandgap reference and the low power characteristics of the sub-threshold bandgap reference. However, a reliable integrated solution is required with fault detection capability for the sensor network connected to the system.

In this work, a secured design for a temperature sensor is proposed to address the security issues affecting the BTMS of EVs. The proposed architecture consists of two secure units, one unit (transducer) sensing the temperature in a wide range and detecting fault attacks [19], and another unit leveraging a statistical method to generate an anomaly detection signal. Our secure architecture can identify and isolate the compromised sensing node based on these two secure units. This secured architecture provides a low overhead design with a significant detection rate compared to existing works at the circuit level, ensuring the run-time thermal sensor data. The main contributions of this work include:

(1) We propose a secured temperature sensor design to address the security issues in sensors. More specifically, the proposed sensor has two secured units, one unit (transducer) sensing the temperature in a wide range and detecting fault attacks and another unit leveraging a statistical method to generate an anomaly detection signal.
(2) We leverage the principle of temperature compensation to design the transducer unit, which exploits two complementary currents to detect attacks in sensors without a golden reference.
(3) A statistical method is utilized to compare the estimated sensor data and the real-time data and then detect anomalies in the signal conditioning unit.
(4) The proposed secure sensor can identify and isolate the fault attacks with a low overhead design compared to existing works at the circuit level.

The rest of the work is organized as follows. Section 2 presents different attack scenarios on the sensor architecture. In Section 3, the defense methods of our proposed sensor architecture are proposed. In Section 4, the performance evaluation of our sensor is provided with the proposed security architecture. In Section 5, the limitations of this work are discussed with an explanation of possible future work. This work is concluded in Section 6.

## 2. Attack Scenarios on Temperature Sensors

**Figure 2:**
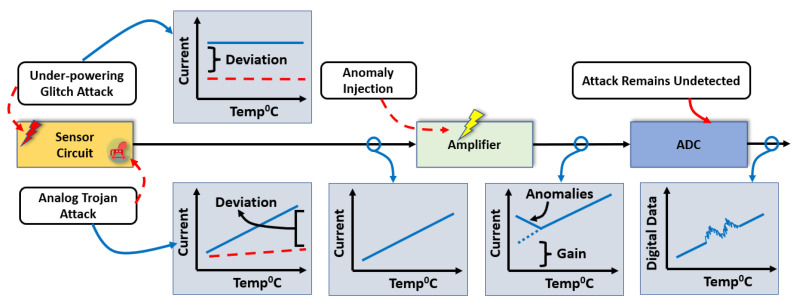
Attack scenarios in sensor architecture.

Since the BTMS’s operations rely substantially on the data collected by temperature sensors, a sensor error can compromise a battery’s performance and pose serious safety hazards [20]. In a traditional sensor system, an analog sensor signal from the sensor element goes to the amplifier circuit. An amplifier circuit converts the analog sensor signal with a certain amount of gain to reach the input range of analog to digital converter (ADC). There are multiple nodes open for the attackers to jeopardize the analog sensor signal shown in Figure 2. The attackers can attack both the sensor and the amplifier circuits. An under-powering glitch attack can be applied by attaching an additional faulty power source to the sensing unit’s supply pin (VDD) to pull down its nominal supply voltage with a short time interval. As under-powering glitches are a significant source of power supply noise, the magnitude and the duration of the glitches are the critical factors that determine the severity of the attacks [21]. The short-duration glitches bring the transistors in their linear region from the saturation [22].

On the other hand, an analog Trojan [23] leverages the analog components in a system to induce intentional noise, which is hard to differentiate from environmental noise. In addition, dynamic analog hardware Trojans [24] have multiple operating modes, which could be triggered accidentally or intentionally. The Trojans [25] add a specific malicious signal as noise which can break the balance of the sensing unit.

An amplifier circuit is also vulnerable due to having an opportunity to access the components physically. When an amplifier is compromised due to any anomalous signal or attack, the ADC will receive a faulty amplified sensor signal. A range of sensitive temperatures (lower or higher) can be specifically targeted using a temperature-dependent trigger-based circuit. Such an attack could be conducted by a covert anomaly injection technique. Two different types of thermal resistors can be used as a voltage divider circuit to generate a trigger signal which activates only in selected temperature ranges by attackers. We use this trigger signal to activate only in the range of low temperatures. The specific range of temperatures (lower than 5 ${}^{{}^{\circ}}$C) has been targeted from the thermally vulnerable nodes of lithium-ion batteries. The trigger signal activates the anomaly injection path to the amplifier circuit. This attack injects anomalous data into the amplified signal to compromise that particular temperature range, keeping the other regions of operations unaffected.

## 3. Proposed Secure Sensor Architecture

### 3.1. Overview of Proposed Sensor

**Figure 3:**
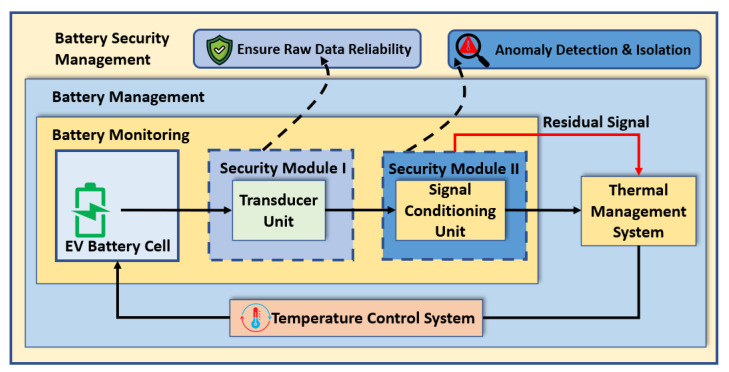
Concept of secure temperature sensor architecture.

A sensor network can be affected by various attacks, intentionally or unintentionally. To mitigate the possible attacks mentioned in Section 2, we propose a sensor architecture in which a transducer and a signal conditioning unit are added to an existing sensor, as shown in Figure 3. The transducer unit has several sensing elements that provide reliable raw data to the signal conditioning unit. Before processing the raw data in the signal conditioning unit, we ensure the resilience of the transducer unit in security module I. We propose an attack-resilient temperature sensor that can thwart attacks at the transducer level. This attack-resilient transducer unit can sense the ambient temperature reading from the EV’s battery pack and detect intentional and unintentional attacks. However, an unprotected signal conditioning unit can still be affected even with an attack-resilient transducer unit. Therefore, the signal from the transducer must be processed in a secure way to detect and isolate the faults. The proposed method in security module II generates a residual signal to indicate anomalous data. Based on the detection of anomalous data, the isolation process is applied either in the transducer or in the signal conditioning unit. Thus, the fault detection and isolation process leads to a secure temperature sensor architecture which ensures reliable sensor data. Thus, the BTMS operates the temperature control system after analyzing the reliable sensor data.

### 3.2. Attack-Resilient Transducer Unit

**Figure 4:**
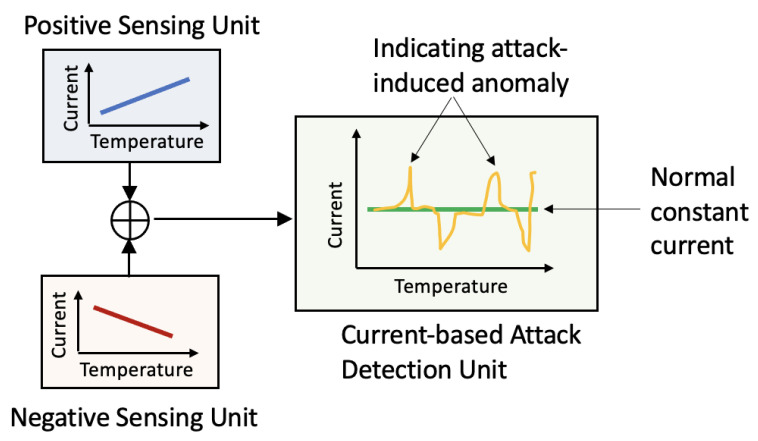
Concept of the proposed sensor for current-based attack detection.

The attack-resilient attribute of the proposed secure transducer is achieved by integrating two sensing circuits, which are either positively or negatively proportional to the temperature. As shown in Figure 4, the current-based attack detection unit integrates the output from the positive and negative sensing units. Then, the detection unit examines the current vibration to generate a warning signal. Without sensor attacks, our current-based attack detection unit maintains a constant current. In contrast, current glitches indicate an attack on the transducer unit.

**Figure 5:**
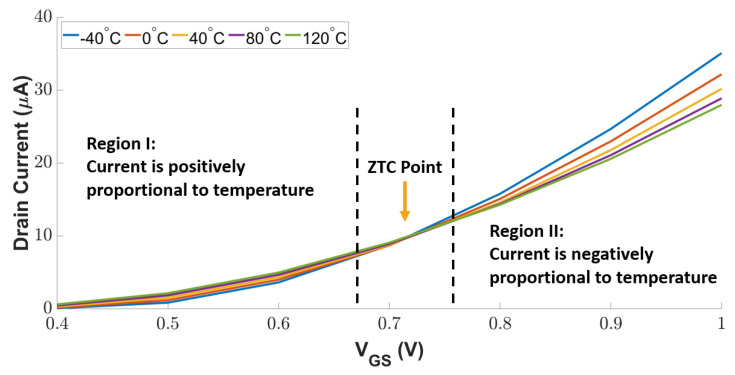
I–V characteristics of diode-connected MOSFET.

To be compatible with the other digital modules, we used MOSFETs to implement the positive and negative sensing units in Figure 4. A diode-connected MOSFET operates in its saturation mode, in which the drain current is determined by the applied gate-source voltage $V_{GS}$. As temperature variation changes the transistor threshold voltage, the drain current varies with temperature. Figure 5 depicts the impact of temperature and $V_{GS}$ on the drain current of a diode-connected MOSFET. As can be seen, the drain current decreases with the increasing temperature in Region I (i.e., positively proportional relationship); in contrast, a higher temperature leads to more drain current in Region II (i.e., negatively proportional relationship). Between Region I and Region II, a Zero Temperature Coefficient (ZTC) point exists, at which the transistor’s mobility and threshold voltage are mutually compensated in a certain range of temperatures. Thus, the transistor is resilient against temperature variation.

**Figure 6:**
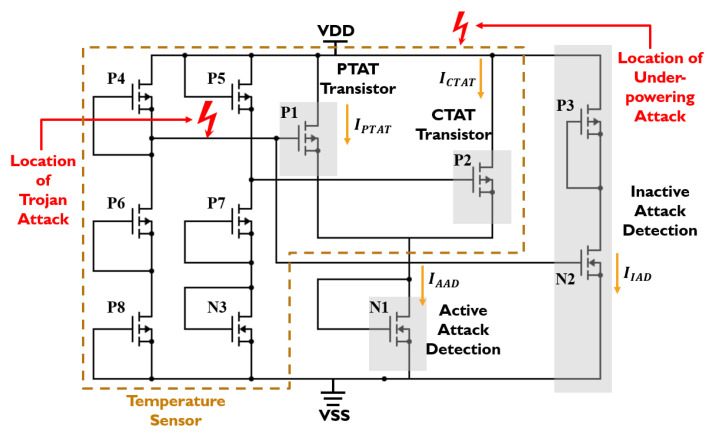
Circuit diagram for the proposed temperature sensor.

We leverage this complementary current–temperature dependency to design an attack-resilient temperature sensor circuit, as shown in Figure 6. The transistors P1 and P2 operate as two sensors, the current of the P1 transistor is positively proportional to the absolute temperature (PTAT) and the current of the P2 transistor is complementarily proportional to the absolute temperature (CTAT). The PTAT and CTAT transistors have a linear dependency between carrier concentration and temperature. The transistor N1 combines the currents from PTAT and CTAT to form a constant current reference for active attack detection. The constant current reference holds some variation with temperature. The impact of temperature on current variation is minimized by tuning the MOSFETs aspect ratio. To detect inactive attacks, an extra branch composed of the P3 and N2 transistors is introduced to draw a significant amount of current, even if there is a slight change in the voltage. The rest of the transistors in the sensing circuit are responsible for providing the proper gate voltages for the PTAT and CTAT transistors, as well as active and inactive attack detection circuits. The proper $V_{GS}$ for the PTAT and CTAT transistors is determined from the observation shown in Figure 5. Furthermore, we exploit the principle of temperature compensation [26] to reduce the dependency between $V_{GS}$ and temperature.

### 3.3. Anomaly Detection Unit

**Figure 7:**
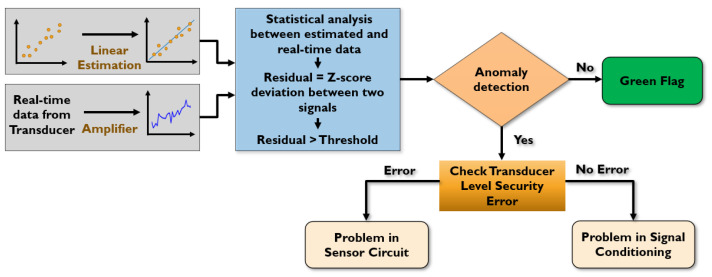
Anomaly detection flowchart in signal conditioning unit.

The signal conditioning unit will be vulnerable to security attacks. To address this issue, we propose an anomaly detection and mitigation method. The flowchart of our method is shown in Figure 7.

We leverage an estimation theory to determine the estimated signal from the sensor performance. Since the temperature readings from our sensors are linearly correlated, a linear estimation theory is applied to generate a linear model. The estimated sensor data and real-time sensor data are analyzed using a statistical method called Z-score. The Z-score deviation between the two signals generates a residual signal compared with a threshold value. A threshold with a certain acceptable range of inaccuracy (3-sigma deviation) is required to produce an error flag. We can determine the threshold by analyzing a different range of white noise amplitude. As a 3-sigma deviation covers 99.73% noise samples, our method considers almost all the noises in the signal without interrupting the sensor readings. If the residual signal exceeds the threshold, our method detects any anomalies in the sensor data. To isolate the faulty unit, we need to investigate the transducer level reliability from the variation of the current-based attack detection unit. If errors are found in the transducer unit, this unit must be isolated. Otherwise, the amplifiers in the signal conditioning unit are compromised and need to be replaced.

#### 3.3.1. Sensor Reading Estimation

The readings from the temperature sensor transducer (PTAT or CTAT) are linear with respect to temperature. To fit the linear readings of sensor data, a contemporary least square method [27] is developed to perform the linear estimation. Assume the sensor data can be expressed as Equation (1).

(1) $$y_{i}=mx_{i}+c$$

where *i* = 1, 2, 3, ⋯, n. Equation (1) is a straight line equation where m is the slope of the straight line, *c* is the y-intercept, and *x*${}_{i}$ and *y*${}_{i}$ are the coordinates of the x–y axis. We assume the *x*${}_{i}$ and *y*${}_{i}$ are the temperatures and corresponding sensor readings, respectively. The square of Equation (1) is employed and added for all the variables to calculate the Squared Errors (SSE). The (SSE) of this linear equation is shown in Equation (2).

(2) $$SSE=\sum_{i=0}^{n}{\left(y_{i}-mx_{i}-c\right)}^{2}$$

To minimize the difference between the estimated and real-time sensor data, a partial derivative of SSE is required with respect to m and c. The derivatives are set to zero to build derivative equations. After solving those derivative equations, we can determine the value of m and c by using Equation (3) and Equation (4), respectively.

(3) $$m=\frac{\sum_{}^{}x_{i}y_{i}-\sum_{}^{}x_{i}\sum_{}^{}y_{i}}{\sum_{}^{}x_{i}^{2}-{\left(\sum_{}^{}x_{i}\right)}^{2}}$$

(4) $$c=\frac{m\sum_{}^{}x_{i}-\sum_{}^{}y_{i}}{n}$$

Now, we can obtain the estimated output of the sensor data by placing the value of *m* and *c* to Equation (1).

#### 3.3.2. Sensor Anomaly Detection

We propose to use a residual signal to detect the sensor reading anomalies from the signal conditioning unit. We generate the residual signal via a statistical model called Z-Score [28], which is a function of the mean and standard deviation of the estimated signal.

The estimated signal is denoted by y${}_{i}$, and the real-time signal from the transducer is denoted by. The mean of the estimated and real-time data are expressed in Equation (5) and Equation (6), respectively.

(5) $$\mu_{estimated}=\frac{1}{n}\sum_{i=1}^{n}y_{i}$$

(6) $$\mu_{real-time}=\frac{1}{n}\sum_{i=1}^{n}\hat{y_{i}}$$

The standard deviation of the sensor estimated and real-time data are calculated from the mean value of the sensor data. The standard deviations of the estimated and real-time data are shown in Equation (7) and Equation (8), respectively.

(7) $$\sigma_{estimated}=\sqrt{\frac{1}{n-1}\sum_{i=1}^{n}{\left(y_{i}-\mu_{estimated}\right)}^{2}}$$

(8) $$\sigma_{real-time}=\sqrt{\frac{1}{n-1}\sum_{i=1}^{n}{\left(\hat{y_{i}}-\mu_{real-time}\right)}^{2}}$$

Now, we calculate the Z-score for all the estimated and real-time sensor data from the previously calculated mean and standard deviation signal. The Z-scores of the estimated and real-time sensor data are denoted by Z${}_{i}$ and, as represented by Equation (9) and Equation (10), respectively.

(9) $$Z_{i}=\frac{y_{i}-\mu_{estimated}}{\sigma_{estimated}}$$

(10) $$\hat{Z_{i}}=\frac{\hat{y_{i}}-\mu_{real-time}}{\sigma_{real-time}}$$

As expressed in Equation (11), the residual signal is the difference between the Z-scores of the estimated and real-time sensor data:

(11) $$Residual=Z_{i}-\hat{Z_{i}}$$

As the residual signal reflects all the system noises, we consider the noise distribution to set a threshold. For a Gaussian distribution of noise signal, 99.73% of noise is distributed within the 3-sigma deviation. Thus, we set the threshold by considering the 3-sigma deviation. When the residual signal oversteps the threshold, an anomaly detection signal will be generated to alarm the system, indicating that there is a temperature reading error in the system caused by attacks. To isolate this problem, the transducer level detection system should be checked. If no fault occurs at the transducer level, then some anomaly attacks happen in the signal conditioning unit.

## 4. Simulation-Based Evaluation

### 4.1. Simulation Setup

**Figure 8:**
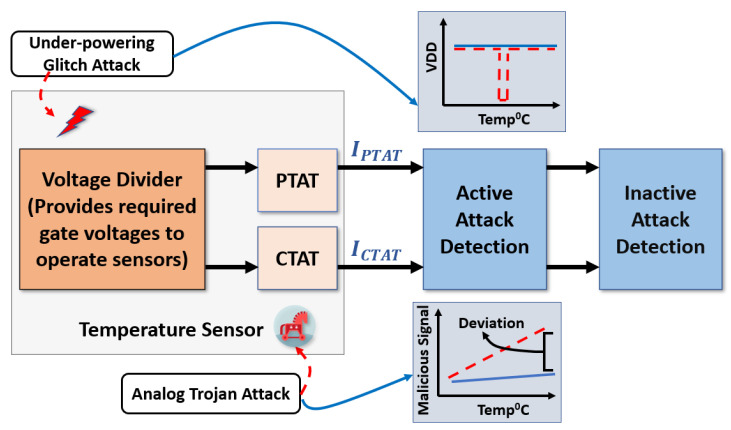
Simulation setup for the proposed sensor transducer.

The proposed sensor was simulated in 180nm CMOS technology. The currents for PTAT, CTAT, active, and inactive attack detection transistors were simulated and measured from the Cadence Virtuoso environment. The nominal supply voltage is 1.8 V. We use NI Multisim software to process the signals coming from the proposed sensor. A voltage source emulated the under-power attack. The analog Trojan [25] was introduced to the sensor to cause intentional fault attacks. The proposed circuit was simulated in Cadence and is shown as a block diagram in Figure 8.

**Figure 9:**
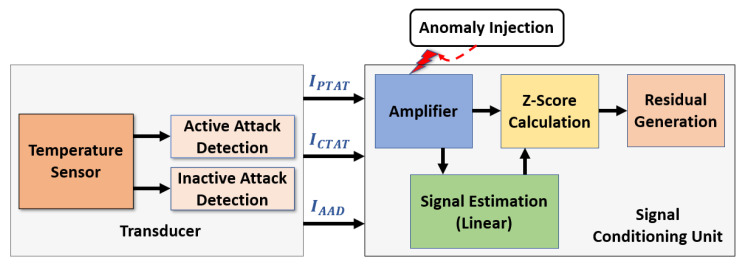
Simulation setup for the proposed sensor with signal conditioning unit.

Furthermore, an anomaly injection circuit was applied in the signal conditioning unit. The setup for the signal conditioning unit is shown in Figure 9. The anomaly injection circuit was triggered to inject anomalous data into the amplifier circuit at a temperature range from −40 ${}^{{}^{\circ}}$C to 5 ${}^{{}^{\circ}}$C by using two thermal resistors as a voltage divider circuit. This voltage divider circuit provides a significant amount of voltage to activate the anomaly injection circuit. The anomalous data are examined using the Z-score calculation and generated residuals by comparing them with the estimated signal.

### 4.2. Key Performance of Proposed Temperature Sensor

**Figure 10:**
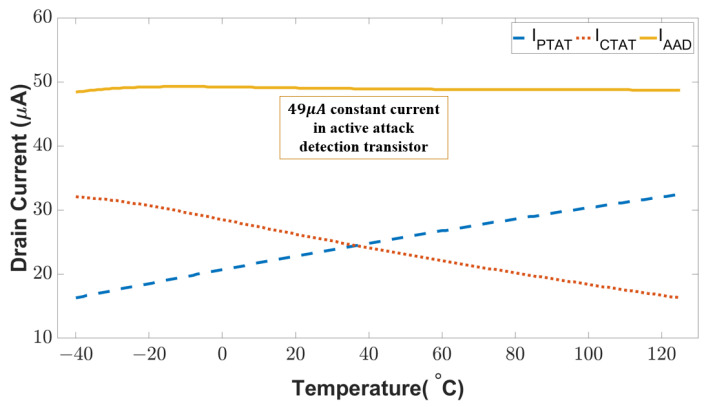
Drain current for the PTAT, CTAT, and active attack detection circuit in fault-free condition.

We first assessed the performance of our sensor in a wide range of temperatures and input frequencies. Figure 10 shows the drain currents of the key transistors in our sensing circuit. As can be seen, I${}_{PTAT}$ is proportional to the temperature. In contrast, I${}_{CTAT}$ is inversely proportional to the temperature. The complementary characteristics of the PTAT and CTAT current enable the sensor to obtain a constant current (I${}_{AAD}$∼49 μA) flowing through the active attack detection transistor regardless of the operational temperature. Any unintentional upset (natural fault) or intentional disturbance (fault attack) that breaks the constant current will indicate an anomaly in the sensor. More importantly, this complementary feature is a built-in feature that is not removable. Thus, the proposed circuit can thwart the attack which attempts to bypass or remove the built-in defense mechanism.

The performance of sensors typically varies with the fabrication process. We examined the constant characteristics of the combined current from the PTAT and CTAT sensing branches in three process corner cases (i.e., typical-typical, fast-fast, slow-slow). The current deviation σ is a metric (Equation (12)) to indicate the impact of process variation on attack detection sensitivity.

(12) $$\sigma =\frac{I_{realtime}-I_{ref}}{I_{ref}}$$

In which $I_{realtime}$ is the instant current measurement and $I_{ref}$ is the constant drain current of the active attack detection transistor in the sensor. As shown in Table 1, the variation caused by the different process corners is negligible. The variation of the sensor performance is less than 2.08% for the typical corner and 3.83% for the fast corner. The variation in the slowest corner is slightly higher than in the typical and fast corners because the PTAT transistor produces less current.

#### 4.2.1. Power Supply Rejection Ratio

**Figure 11:**
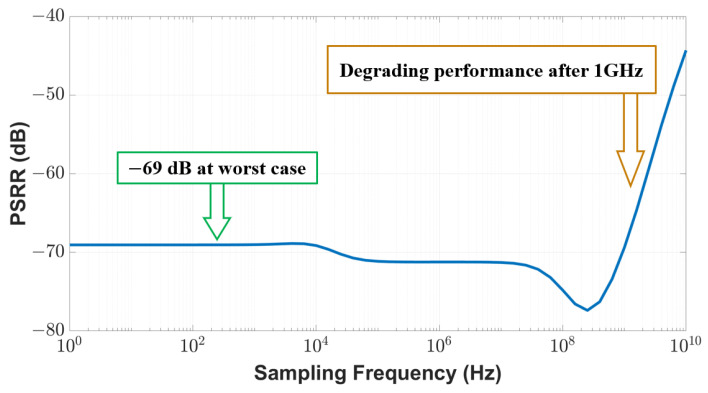
PSRR of the proposed sensor.

Every electronic system with multiple loads creates ripples in the output node. If not managed, the voltage ripple in a sensor circuit could affect the accuracy of the sensor. We adopted the Power Supply Rejection Ratio (PSRR) as a metric to quantitatively measure the ripple effect. The definition of PSRR is expressed in Equation (13).

(13) $$PSRR=-20\log_{10}\left(\frac{V_{Supply}}{V_{Out}}\right)$$

where $V_{Supply}$ is the supply voltage connected to the proposed temperature sensor and $V_{Out}$ is the output voltage at the sensor node. A higher PSRR (absolute value) indicates better sensor performance. As shown in Figure 11, our sensor achieves a PSRR of −69 dB within the 1 GHz range, which is 11% better than the performance reported in the existing work [29]. As the sampling frequency goes beyond 1 GHz, the PSRR performance of our sensor will degrade.

#### 4.2.2. Resilience against Under-Powering Attack at Transducer Level

**Figure 12:**
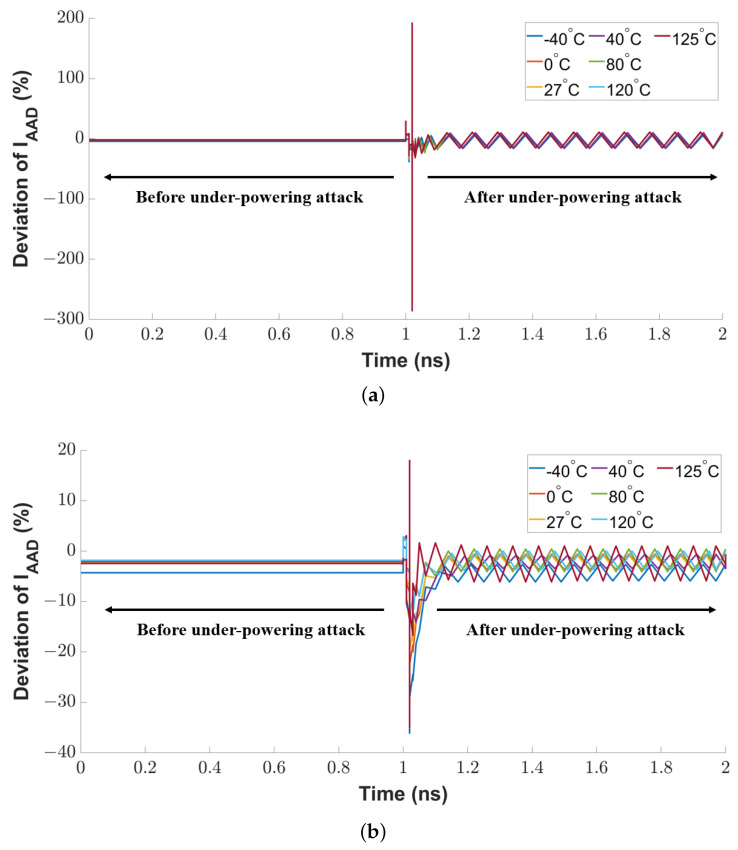
Sensitivity of proposed sensor circuit against under-powering voltage glitch attack on (**a**) PTAT transistor and (**b**) CTAT transistor in the sensor.

As explained in Section 4.2, the current of the active attack detection transistor in the proposed sensor remains constant in normal conditions. Any vibration on that current can indicate an anomaly in the sensor. We use the current deviation as a metric to evaluate the attack detection sensitivity. At first, we conducted an under-powering attack on the sensor circuit by attaching an additional power source to the supply pin (VDD) of the CTAT or PTAT transistor to pull down its nominal supply voltage from 1.8 V to 0.8 V with a time interval of 100 ps. As shown in Figure 12a, there is a sharp spike at the rising edge of the voltage glitch because the attack breaks the stable conductive channel between the drain-to-source terminal of the sensing MOSFET. The peak vibration range of the spike is 284.9%. After the attack duration, the residual vibration (in a range of 42.85%) remains in the current deviation.

We repeated the same evaluation technique by injecting a voltage glitch into the CTAT transistor’s supply voltage pin. As shown in Figure 12b, the attack also induces a noticeable transition at the rising edge of the voltage glitch. The maximum sensitivity is 36.1%, and the impact of the temperature variation on the current deviation is 23%.

#### 4.2.3. Resilience against Analog Trojan Attack at Transducer Level

**Figure 13:**
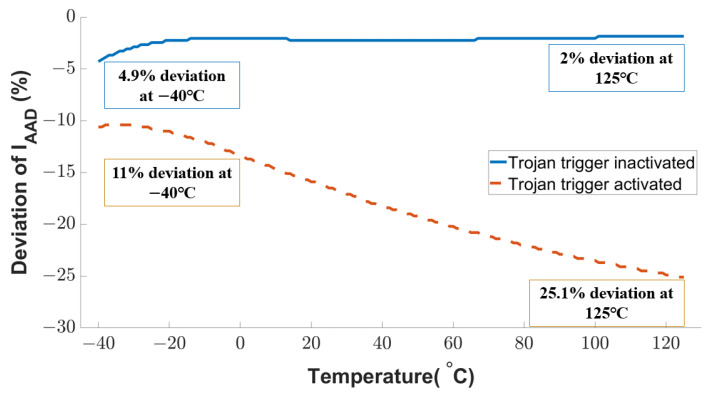
Sensitivity of proposed sensor circuit against Trojan attack.

Another intentional fault attack examined in this work is analog Trojan [23]. The Trojan payload adds an extra voltage to the sensing transistor’s gate voltages, and an external source or clock voltage triggers the analog Trojan. The Trojan-induced current deviation of the active attack detection transistor is shown in Figure 13. If the Trojan is not activated, the current variation is negligible (4.9% at the temperature of −40 ${}^{{}^{\circ}}$C and 2% as the temperature increases to 125 ${}^{{}^{\circ}}$C). Once the analog Trojan is triggered, the current deviation starts from 11% and reaches up to 25.1%. The temperature plays an important role here because a higher temperature results in higher mobility of the charge carriers. Compared to the Trojan inactive mode, the current deviation due to the Trojan activity will be 2.2× at −40 ${}^{{}^{\circ}}$C and 12.6× at 125 ${}^{{}^{\circ}}$C. This substantial current deviation can be used to detect Trojans effectively.

**Figure 14:**
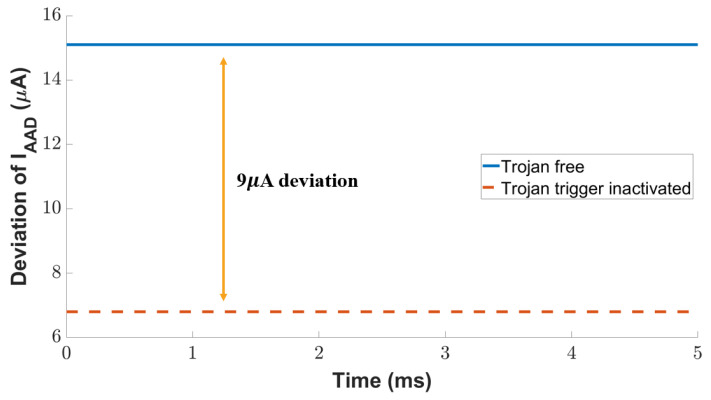
Detection of analog Trojan in the inactive state.

We further exploit the current of the inactive attack detection branch to detect the Trojan even if it is not activated. The diode-connected load in the inactive attack detection circuit draws a large current with a small increase in voltage change. As shown in Figure 14, there is a current difference of 9 µA between the Trojan-free and Trojan-inactive scenarios. The current reduction caused by the inactive Trojan is 55% variation with the golden circuit current, which is 11× to the current monitored by the active attack detection transistor discussed above. In summary, the two current monitoring branches in our attack-resilient sensor provide a high sensitivity against the Trojan, no matter whether it is active or not.

### 4.3. Reliability against Anomalous Data in Signal Conditioning Unit

**Figure 15:**
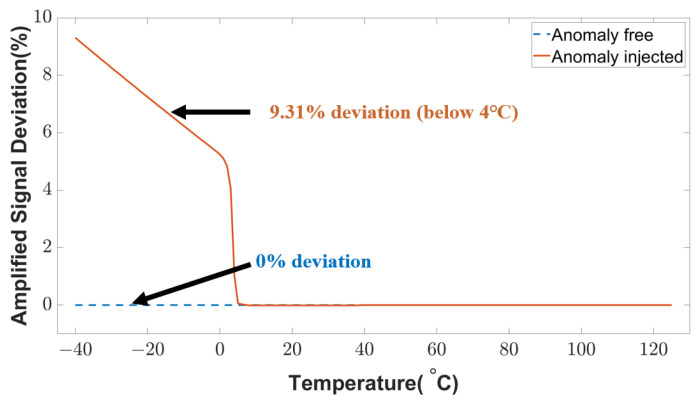
Amplified sensor signal deviation under anomalous conditions.

The signal conditioning unit refers to an amplifier circuit that introduces a certain gain to the signal from a transducer unit. An attacker can inject anomalies into this amplifier unit to modify the original data. This attack could lead to faulty sensor data at the user level. The anomaly-injected and anomaly-free conditions are shown in Figure 15. When the fault is not triggered, the amplified signal deviation is close to 0%. However, the deviation goes up to 9.31% in the case of triggered fault at low temperatures (below 4 ${}^{{}^{\circ}}$C). Any autonomous temperature-dependent system will receive an anomalous signal in this low-temperature region that deceives the temperature control mechanism and creates malfunction in the application.

#### 4.3.1. Estimated Sensor Data

**Figure 16:**
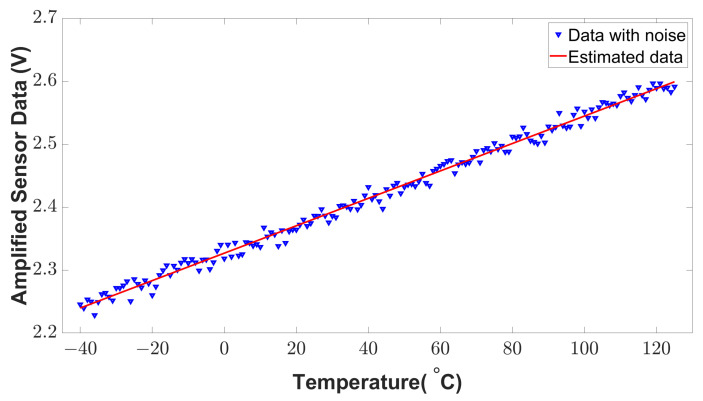
Estimated sensor data using the least square method.

To detect the anomalies from Figure 15, the estimation theory discussed in Section 3.3.1, is applied to the sensor’s original raw data. As the characteristic of the sensor data is linear, we used the least square method to estimate the sensor data with curve fitting. In real applications, the sensor data are noisy due to environmental factors. Therefore, we introduced some environmental noise (white noise) to the sensor data to validate the estimated data. The estimated data and noisy data are shown in Figure 16. As is shown, the estimated sensor data are very close to the sensor’s historical data. To measure the accuracy of the estimation, R-square is calculated from the estimated and historical data. According to the reference of regression analysis evaluation [30], a higher R-square value on the scale of 0 to 1 represents a better correlation between the estimated and historical data. From the simulated result, an R-square value of 0.990146 is found which represents the higher accuracy of estimation.

#### 4.3.2. Anomaly Detection Capability

**Figure 17:**
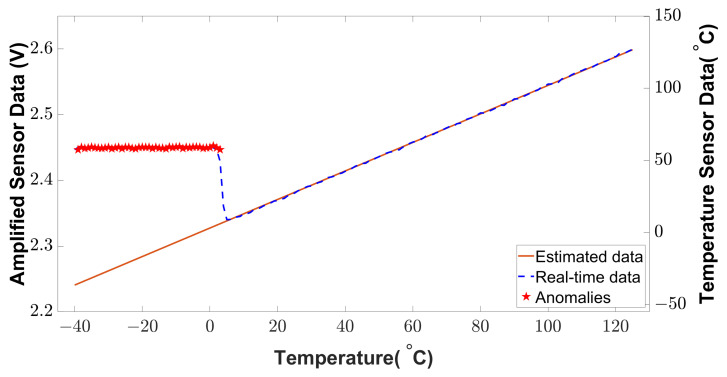
Anomaly detection in low-temperature region.

We applied a statistical method (Z-score) to detect the anomalies from the real-time sensor data. This method can detect anomalies at low temperatures. The anomaly detection from the signal conditioning unit is shown in Figure 17. The BTMS receives the alarm signal for anomaly detection to isolate a certain temperature sensor module. As shown in Figure 17, the anomaly injection changes the lower temperature readings (−40 ${}^{{}^{\circ}}$C to 4 ${}^{{}^{\circ}}$C) to a higher temperature (around 50 ${}^{{}^{\circ}}$C). This faulty temperature reading deceives the BTMS to operate in lower temperatures. When anomalies are detected, the alarm alerts the BTMS of an EV. In this way, the BTMS will isolate a specific sensor module according to the alarm.

**Figure 18:**
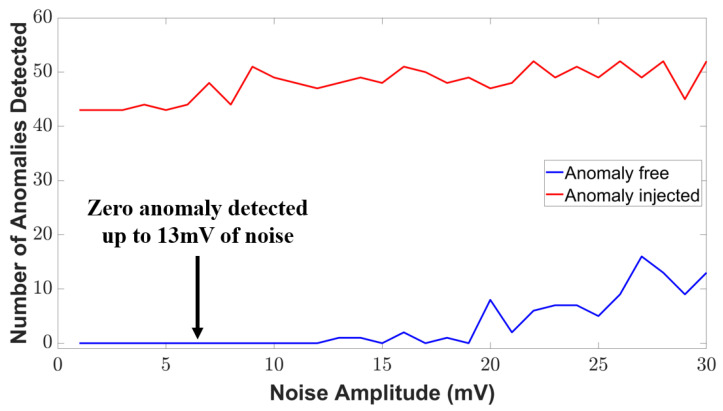
Variation in the detection signal with noise amplitude.

To evaluate the detection signal, we used a metric named anomaly detection rate (*ADR*), defined by Equation (14).

(14) $$ADR=\frac{NDC}{NR}$$

where *NDC* represents the number of anomaly points detected correctly and *NR* represents the number of real anomaly points. Our case study shows that the anomaly detection rate is about 97.73% found from the signal conditioning unit. The detection rate has been significantly increased compared to our reference works [14,31,32]. However, all the electronic unit, such as the transducer unit, has some environmental noise [33]. These noises are being amplified in the signal conditioning unit, which can jeopardize the secured architecture and send false alarms to the BTMS. As a result, we consider the noise magnitude in our simulation to examine the noise tolerance of our method. The variation in anomaly detection with the noise is shown in Figure 18. As can be seen, zero anomalous data are detected up to 13 mV of the noise amplitude in the anomaly-free condition. We observe some anomalous detection after the 13 mV noise amplitude which defines the noise tolerance level of our proposed security method in signal conditioning units. If the noise level exceeds more than 13 mV, this method might give a false alarm and mislead the BTMS.

### 4.4. Quantitative Analysis of Hardware Overhead and Features

The comparison of our transducer circuit’s overhead and features with some existing work is presented in Table 2. We analyzed the sensor performance quantitatively with reference works in terms of temperature range, power consumption, PSRR, and attack detection capability metrics. The work [29] leverages a CTAT voltage to generate a temperature sensing unit with a PSRR of −62 dB. A sub-threshold MOSFET-based temperature sensor [34] working in the temperature range of −55 to 105 ${}^{{}^{\circ}}$C consumes a power of 48 μW and obtains a PSRR of −60 dB. This method [34] improves the sensitivity by adding a simple amplifier consisting of two MOSFETs powered by a simple two-stage regulator. A transistor-based current-mode thermal sensor [15] uses FinFET technology and a single-element remote-sensing technique and consumes a power of 50μW in a wide range of temperatures. A fully integrated temperature sensor [17] utilizes the difference between the PTAT current and reference current for sensing over a temperature range of −40 to 100 ${}^{{}^{\circ}}$C. In addition, this existing method [17] incorporates process variation compensation with 264 μW power consumption in 180 nm technology. A complimentary current-mode approach [16] utilized a single feedback loop to design a compact thermal sensor with a PSRR of 2 ${}^{{}^{\circ}}$C/V (−43.98 dB). This compact design [16] consumes around 32% less power compared to our design. However, our proposed method has 36.25% higher PSRR than [16]. A bandgap reference voltage source for smart grid sensor system-on-chip [18] consumes power of 65 μW between −40 to 85 temperature ranges with −78 dB of PSRR. Though this method [18] has improved PSRR than our proposed method, the power consumption is 58% is higher than our proposed method. Compared to the minimum power consumption from other references [34], the proposed sensor consumes 17% less power and 11% higher PSRR (absolute value) because of security measures with less complexity and low overhead. The most important feature of our proposed sensor is sensor-level attack detection. This is not available in the closely related sensors in [15,16,17,18,29,34].

On the other side, our proposed anomaly detection in the signal conditioning unit has been compared with some of the reference work [14,31,32]. The work [32] has a fault estimation error for the temperature sensor of 5% after using the particle filters for a stable residual. A regressive model-based fault detection method [31] has around 80% detection rate for the sensor bias error. The work [14] utilized a certain number of statistical methods for fault detection and obtained an 83.96% fault detection rate. In the case of our work, we revealed both the transducer and signal conditioning units’ resilience with an anomaly detection rate of 97.73%, which is 17.73% and 13.77% higher in detection rate than [31] and [14], respectively.

## 5. Discussion and Future Work

Our work demonstrated a secure temperature sensor design in the transducer and signal conditioning unit. The transducer produced current-based signals with respect to temperatures. These signals have been used to analyze the sensor performance by comparing with closely related sensors [15,16,17,18,29,34]. In addition, the performance of anomaly detection has been compared with other reference works [14,31,32]. The prior papers [14,31,32] are dedicated for detecting faults based on different types of statistical methods. As discussed in Section 4.4, the performance of the proposed sensor and anomaly detection achieves low power, high PSRR, and fault detection capability at the circuit level. Furthermore, a process-temperature variation has been analyzed to show the reliability of the proposed method. However, security threats and attack techniques are evolving rapidly. Circuit-level security techniques are required to be updated and maintained regularly to counter emerging threats effectively. In addition, the scope of the circuit-level security measures is bounded to only protecting the sensor’s hardware. Though our proposed sensor provides a solid foundation for security and reliability, other potential vulnerabilities, such as software-based attacks and network-based threats, are not addressed in this paper. Multi-layered defense methods should be utilized in comprehensive security strategies to address various potential threats. In future work, we will implement the prototype of the proposed circuit to consider more attack scenarios and measure the performances of the prototype circuit. As sensor performance varies with fabrication materials and its operational environment, we will conduct physical experiments and put the proposed sensor prototype in the context of BTMS.

## 6. Conclusions

To secure raw data of the sensors, most industries require an attack-resilient sensor architecture. Instead of using high-overhead system-level solutions, this work proposes two separately secured units (transducer and signal conditioning) of a temperature sensor in a circuit-level design to thwart the attack that attempts to compromise sensors. The proposed transducer design uses two complementary sensor current properties to produce a constant current reference, enabling it to recognize active and passive attacks. The sensor transducer was subjected to two common attacks—under-powering and hardware Trojan attacks. Moreover, an anomaly detection method has been introduced in the signal conditioning unit of the sensor. The method utilized an estimation theory (linear estimation) and a statistical method (Z-scores) to generate residuals for anomaly detection. Three typical attacks, under-powering, hardware Trojan, and anomalies attacks, were applied to the proposed sensor architecture. Simulation results show that the sensor’s transducer has residual vibration of 42.85%, which is significantly high to detect the under-powering attack. A triggered hardware Trojan leads to a 25.1% deviation from the constant current reference. It can also detect inactivated analog Trojans from a 55% current deviation compared to the fault-free circuit. This architecture also shows a significant anomaly detection rate of 97.73% in the signal conditioning unit with noise tolerance up to 13 mV. In addition, our sensor architecture can operate in a wider temperature range and achieve an 11% higher PSRR (absolute value) than the existing sensors. In future work, we will implement the prototype of the proposed sensor and deploy it to a physical BTMS system. The impact of environmental noises and other security attacks will be considered in our assessment in the future.

## Figures and Tables

**Table 1:** Current deviation σ of the proposed sensor designed with different process corners at different temperatures.

Process/Temp	−40 ${}^{{}^{\circ}}$C	0 ${}^{{}^{\circ}}$C	40 ${}^{{}^{\circ}}$C	80 ${}^{{}^{\circ}}$C	125 ${}^{{}^{\circ}}$C
Typical-Typical	2.08%	−0.20%	0%	−0.20%	−0.41%
Fast-Fast	−3.37%	−1.38%	0.61%	2.30%	3.83%
Slow-Slow	10.31%	1.43%	−0.57%	−2.57%	−4.87%

**Table 2:** Comparison of overhead and features with existing works.

Sensors	Analysis Setup	Temperature Range (${}^{{}^{\circ}}$C)	Power (μW)	Technology (μm)	PSRR (dB)	Attack Detection Capability
ISSCC’18 [15]	Measured	−30 to 120	50	0.022	-	No
VLSI-DAT’21 [16]	Measured	−20 to 125	27.5	0.07	−43.98	No
Sens. J.’16 [17]	Measured	−40 to 100	264	0.18	-	No
IMOC’17 [29]	Simulated	−20 to 120	-	0.09	−62	No
TCAS-II’13 [34]	Measured	−55 to 105	48	0.09	−60	No
ICCECE’23 [18]	Simulated	−40 to 85	65	0.055	−78	No
**This work**	**Simulated**	**−40 to 125**	**41**	**0.18**	**−69**	**Yes**

## Abbreviations

The following abbreviations are used in this manuscript:

EV	Electric Vehicle
BMS	Battery Management System
BTMS	Battery Thermal Management System
PTAT	Proportional to Absolute Temperature
CTAT	Complementary to Absolute Temperature
ZTC	Zero Temperature Coefficient
ADC	Analog to Digital Converter
PSRR	Power Supply Rejection Ratio
